# Reduced White Matter Integrity in Antisocial Personality Disorder: A Diffusion Tensor Imaging Study

**DOI:** 10.1038/srep43002

**Published:** 2017-02-22

**Authors:** Weixiong Jiang, Feng Shi, Huasheng Liu, Gang Li, Zhongxiang Ding, Hui Shen, Celina Shen, Seong-Whan Lee, Dewen Hu, Wei Wang, Dinggang Shen

**Affiliations:** 1 Department of Radiology, The Third Xiangya Hospital, Central South University, Changsha, Hunan 410013, China; 2 Department of Information Science and Engineering, Hunan First Normal University, Changsha, Hunan 410205, China; 3 Department of Radiology and BRIC, University of North Carolina at Chapel Hill, NC, USA; 4 College of Mechatronics and Automation, National University of Defense Technology, Changsha, Hunan 410073, China; 5 Department of Brain and Cognitive Engineering, Korea University, Seoul, Republic of Korea

## Abstract

Emerging neuroimaging research suggests that antisocial personality disorder (ASPD) may be linked to abnormal brain anatomy, but little is known about possible impairments of white matter microstructure in ASPD, as well as their relationship with impulsivity or risky behaviors. In this study, we systematically investigated white matter abnormalities of ASPD using diffusion tensor imaging (DTI) measures: fractional anisotropy (FA), axial diffusivity (AD), and radial diffusivity (RD). Then, we further investigated their correlations with the scores of impulsivity or risky behaviors. ASPD patients showed decreased FA in multiple major white matter fiber bundles, which connect the fronto-parietal control network and the fronto-temporal network. We also found AD/RD deficits in some additional white matter tracts that were not detected by FA. More interestingly, several regions were found correlated with impulsivity or risky behaviors in AD and RD values, although not in FA values, including the splenium of corpus callosum, left posterior corona radiate/posterior thalamic radiate, right superior longitudinal fasciculus, and left inferior longitudinal fasciculus. These regions can be the potential biomarkers, which would be of great interest in further understanding the pathomechanism of ASPD.

Antisocial personality disorder (ASPD) involves behavioral impairments that include poor self-control ability, impulsivity, aggression, and callous-unemotional traits. Individuals with ASPD are more prone to criminal behaviors than the general population. Fazel and Danesh found that 47% of male prisoners were diagnosed with ASPD in worldwide prison systems^1^. Epidemiological studies also reported a prevalence of 2–3% in the general population, with 3% men and 1% women^2^.

Emerging neuroimaging research suggests that ASPD is linked with abnormal brain anatomy. Raine *et al*. found that the prefrontal gray matter volume in ASPD was reduced by about 11%, in comparison to that of the control group^3^. Gray matter volume deficits in the orbitofrontal cortex and dorsolateral prefrontal cortex were also found in studies of antisocial adults^4^. Reduced gray matter volume in the medial and lateral temporal regions was also revealed in ASPD^5^,^6^,^7^. In accord with these findings, studies using diffusion tensor imaging (DTI) found reduced fractional anisotropy (FA) values in the uncinate fasciculus that connects limbic system regions in the temporal lobe to the orbitofrontal cortex^8^,^9^. Based on this evidence, theories that suggest that fronto-temporal abnormalities are associated with antisocial behavior disorders are put forward^10^ and certified continually^9^,^11^,^12^.

Despite many studies on antisocial individuals, the underlying neural pathophysiology of ASPD remains largely unclear, especially in possible impairments of white matter microstructure in ASPD, as well as their relationship with impulsivity or risky behaviors. Moreover, existing DTI studies on ASPD mostly focus on the analysis of FA and/or mean diffusivity (MD). Here, FA is a scalar value that describes the degree of anisotropy of a diffusion process, and MD describes the magnitude of water molecule movement, independent of direction. However, using FA and MD may not be sufficient for investigating specific axonal or myelin abnormalities^13^. For example, identical FA value may comprise different combinations of axial and radial diffusivities. In this regard, the diffusivity along the principal axis of the tensor ellipsoid is measured as axial diffusivity (AD), and the diffusivities in the two minor axes are averaged as radial diffusivity (RD). Here, AD measures the axonal integrity and RD is sensitive to myelination^14^,^15^, so they are able to capture different aspects of white matter microstructure abnormality separately. Hence, an investigation of AD and RD could provide additional important information of white matter pathology^16^. To our knowledge, however, there was no investigation about AD and RD indexes in ASPD to date.

In this study, to understand white matter microstructure in ASPD disorders in more depth, we used multiple diffusion tensor measures, i.e., FA, AD, and RD, in a tract-based spatial statistics (TBSS) analysis. We hypothesized that alterations could be detected by using these measures in ASPD, and there is also potential relationship between these microstructural changes and impulsivity or risky behaviors. This study will provide valuable information regarding the abnormal microstructure of ASPD, and also highlight the potential relations between structural changes and impulsivity or risky behaviors in ASPD patients.

## Results

### Abnormal Fractional Anisotropy in ASPD Patients

**Figure 1:**
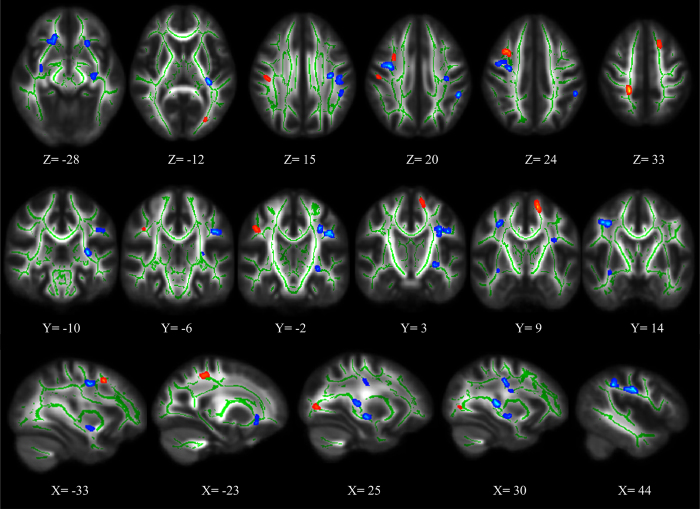
Significant fractional anisotropy (FA) differences in ASPD patients relative to controls (FDR corrected, *p* < 0.05). Blue represents decreased FA value in ASPD patients, and red represents increased FA value in ASPD patients.

Compared to healthy controls (HC), ASPD patients show significant FA differences in several major white matter tracts. Among them, 9 clusters show significant FA decreases in the following tracts (*p* < 0.05, FDR corrected) (Fig. 1, Table 1): right uncinate fasciculus (UNC), left superior longitudinal fasciculus (SLF), bilateral inferior fronto-occipital fasciculus (IFOF), bilateral anterior corona radiate (ACR), right anterior limb of internal capsule (ALIC), left retrolenticular part of internal capsule (RPIC), left superior corona radiata (SCR), left fornix/stria terminalis, and several superficial whiter matter regions, such as the right middle frontal blade, left post-central blade and left parieto-temporal blade. There are also 6 clusters that show significant FA increases (*p* < 0.05, FDR corrected) in the right corticospinal tract, right SLF, left IFOF, and some superficial whiter matter regions, such as the left superior frontal blade and right middle frontal blade (Fig. 1, Table 1).

### Decreased Axial Diffusivity in ASPD Patients

**Figure 2:**
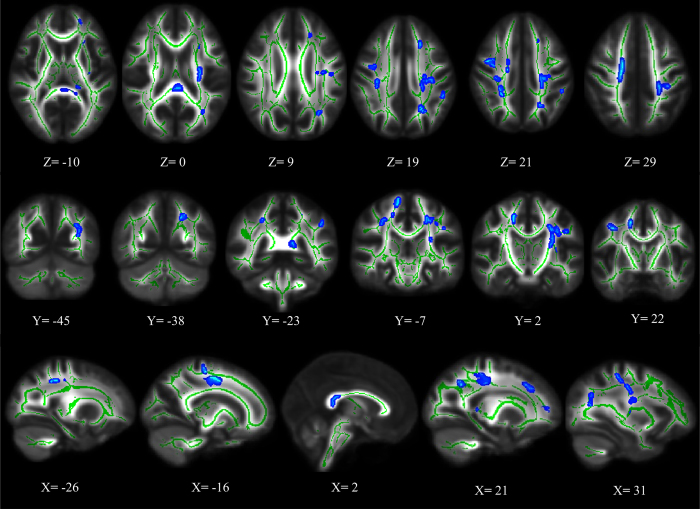
Significant axial diffusivity (AD) deficits in ASPD patients relative to controls (FDR corrected, *p* < 0.05). Blue represents decreased AD value in ASPD patients.

Compared to healthy controls, ASPD patients show significantly lower AD in 17 clusters located in the following tracts (*p* < 0.05, FDR corrected) (Fig. 2, Table 2): bilateral SLF, splenium of corpus callosum (SCC), bilateral SCR, left posterior corona radiate (PCR), left ACR, left anterior limb of internal capsule (ALIC), left RLIC and posterior limb of internal capsule (PLIC), left posterior thalamic radiation (PTR) (including optic radiation), left fornix/stria terminalis, and some superficial whiter matter regions, such as the right precentral blade, right superior frontal blade, left post-central blade, left parieto-temporal blade and left inferior frontal blade. No cluster with significantly higher AD was found in ASPD patients.

### Increased Radial Diffusivity in ASPD Patients

**Figure 3:**
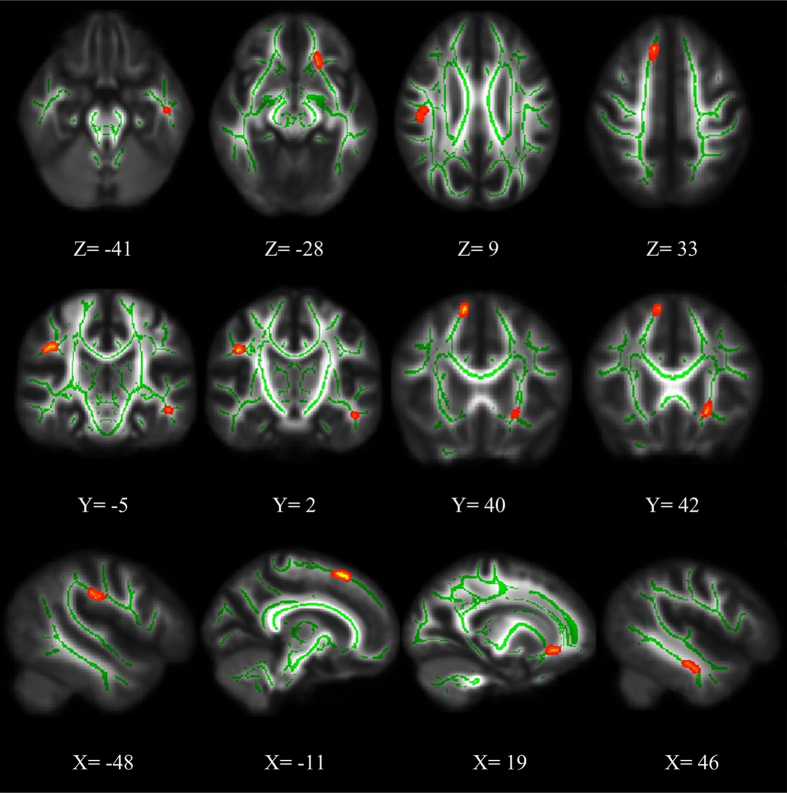
Significant radial diffusivity (RD) deficits in ASPD patients relative to controls (FDR corrected, *p* < 0.05). Red represents increased RD value in ASPD patients.

Compared to healthy controls, ASPD patients show significantly higher RD in 4 clusters of the following tracts (*p* < 0.05, FDR corrected) (Fig. 3, Table 2): right SLF, left ACR, left IFOF, left inferior longitudinal fasciculus (ILF), right superior frontal, and left temporal superficial whiter matter. No cluster with significantly lower RD was found in ASPD patients.

### The overlapping WM areas of abnormal DTI measures in ASPD Patients

**Figure 4:**
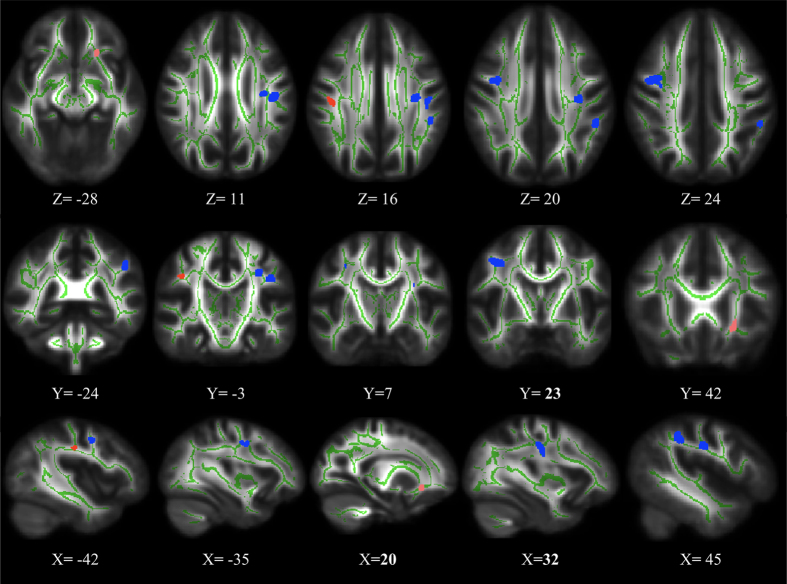
Overlapping WM areas between DTI measures (FA, AD and RD) in ASPD patients relative to controls (FDR corrected, *p* < 0.05). Blue represents decreased FA and AD value in ASPD patients, red represents decreased FA and increased RD value in ASPD patients, and pink represents increased FA and RD value in ASPD patients.

We studied the overlap of abnormal DTI measures in ASPD patients (*p* < 0.05, FDR corrected) and found 6 overlapping clusters in total, as shown in Fig. 4. First, for FA and AD, we found four overlapping areas from the contrast of FA value (ASPD < HC) and AD value (ASPD < HC), which were located in biolateral SLF and left SCR. Then, for FA and RD, we found two overlapping clusters, i.e., one from the contrast of FA value (ASPD < HC) and RD value (ASPD > HC), which was located in the left IFOF; and the other from the contrast of FA value (ASPD > HC) and RD value (ASPD > HC), which was located in the right SLF. At last, for AD and RD, we did not find any overlapping clusters.

### Correlation

**Figure 5:**
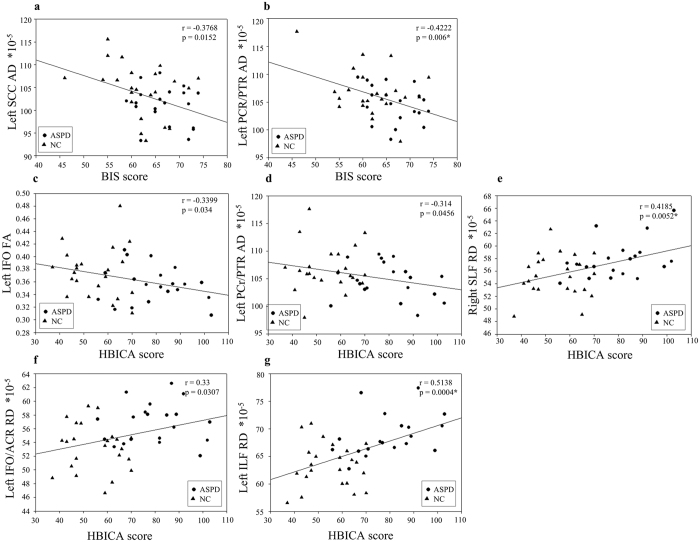
Significant correlation between behavioral scores (BIS score/HBICA score) and DTI measurement: fractional anisotropy (FA), axial diffusivity (AD), and radial diffusivity (RD). *In the top-right of three subfigures indicates *p* < 0.05 after false discovery rate (FDR) correction.

For the average FA, AD, and RD values in each cluster, we first performed correlation analysis with impulsivity, which was measured using the Barratt Impulsiveness Scale (BIS). For both FA and RD values, no significant results were found. For AD values, 2 clusters were correlated negatively and significantly with BIS scores (*p* < 0.05, uncorrected), and located in the splenium of corpus callosum (Fig. 5a) and the left posterior corona radiate/posterior thalamic radiate (Fig. 5b).

Then, we performed correlation analysis between their risky behaviors and DTI measures of each cluster. Their risky behaviors were measured using the health-risk behavior inventory for Chinese adolescents (HBICA). There was one cluster located in the left inferior fronto-occipital fasciculus (Fig. 5c) for FA, and another cluster located in the left posterior corona radiate/posterior thalamic radiate (Fig. 5d) for AD, which were correlated negatively and significantly with HBICA scores (*p* < 0.05, uncorrected). For RD values, 3 clusters were correlated positively and significantly with the HBICA scores (*p* < 0.05, uncorrected) in the following tracts: the right superior longitudinal fasciculus (Fig. 5e), left inferior fronto-occipital fasciculus/anterior corona radiata (Fig. 5f), and left inferior longitudinal fasciculus (Fig. 5g). Note that all these three clusters have decreased RD values in ASPD, compared to normal controls.

We have also applied multiple comparison correction using false discovery rate (FDR), and still found 3 clusters whose DTI measures showed significant correlation with the BIS or HBICA scores (*p* < 0.05, FDR corrected), i.e., in the left posterior corona radiate/posterior thalamic radiate (Fig. 5b), the right superior longitudinal fasciculus (Fig. 5e), and the left inferior longitudinal fasciculus (Fig. 5g).

## Discussion

In this study, we investigated white matter microstructure in ASPD patients using DTI, as well as the correlation between DTI measures and impulsivity or risky behaviors. To better understand white matter involvement in ASPD disorders, we supplemented the FA analysis with the analyses of axial diffusivity (AD) and radial diffusivity (RD), which would help provide more information of white matter pathology^16^. These results together could reveal the abnormal WM microstructure of ASPD disorders comprehensively.

We found decreased FA value in ASPD patients mainly within the right UNC, left SLF, bilateral IFOF, left RPIC, left SCR, and left ACR. We also found AD and RD deficits in several other tracts that were not detected by FA, mainly including SCC, left ALIC, right SLF, right ACR, right SCR, and left EC. It is interesting that AD identified a greater number of ASPD abnormalities, which are not found in FA. Note that AD indices are the most sensitive to axonal structural integrity that may be more related to ASPD microstructural abnormalities in relation to the FA (which measures the overall anisotropy). More interestingly, more regions were found correlated with impulsivity or risky behavior in AD and RD values, including the SCC, left PCR/PTR, right SLF, and Left ACR. These correlations were mainly based on uncorrected results, and thus our discussion was also based on these uncorrected correlations. AD and RD measurements are thought to capture different aspects of white matter abnormality, i.e., reduced AD can reflect abnormalities of the axon structure, while increased RD implicates demyelination^14^.

Right UNC abnormality, with reduced FA, found in our study is consistent with the previous DTI study of ASPD^9^. This finding was also reported in different DTI studies on psychopathy^8^,^11^,^17^. The UNC connects the orbitofrontal cortex and the amygdala, thus impaired regulation of amygdala activity by the orbitofrontal cortex may contribute to behavioral disinhibition in ASPD and psychopathy^18^. The UNC is also the major white matter pathway connecting ventral frontal and anterior temporal cortices^11^, which is considered to play a critical role in social-affective function and decision-making^19^,^20^. These were believed to underlie a host of social, cognitive, and emotion regulation relevant to ASPD, such as moral judgment, empathy, and aggression^21^,^22^. Furthermore, the abnormalities in the UNC were believed to be associated with impairments of conditional associative learning^23^. ASPD disorders demonstrated reversal-learning deficits^24^, which may lead to the perseveration of antisocial behavior and also the high levels of recidivism characteristic of the disorder^9^. On the other hand, the stria terminalis that has abnormal FA and AD indices in our study was believed to be a major output pathway of the amygdala, and was thought to correlate with anxiety in response to threat monitoring^25^. It was also thought to relate with behavioral inhibition by the input from the orbitofrontal cortex^26^. The integrity of the UNC and stria terminalis may be closely associated with particular behavioral traits of ASPD.

In our study, the inferior fronto-occipital fasciculus (IFOF) has decreased FA, AD and increased RD. The IFOF connects the frontal and occipital lobes, and possible damage to this tract may result in visual neglect, due to impaired modulation of the frontal cortex^27^. Furthermore, IFOF is involved in insula connectivity, and both were believed to play an important role in emotional regulation and cognition^28^. FA/AD reduction in the present study was also found in the retrolenticular part of the internal capsule and posterior corona radiate, which carry fibers of the optic radiation. The AD values in ACR/PCR were found to correlate with impulsivity and risky behavior. Reduced microstructural integrity of these tracts may contribute to deficits in facial emotional recognition in ASPD disorders, when recognizing fearful, sad, and surprised expressions^29^. Specially, deficits in fear-processing in antisocial populations may contribute to impaired moral socialization^30^. Overall, the low FA value of the inferior fronto-occipital fasciculus together with the retrolenticular part of the internal capsule and ACR may result in disinhibition, poor decision-making, and an inability to follow the rules in ASPD disorders, as well as create negative symptoms^28^.

The SLF had abnormal FA, AD and RD indices, and also RD values in this region were found to be correlated with risky behavior. These deficits have previously been linked to social information processing impairments. A study that focused on youth at high risk for psychotic illness found FA deficits in the superior longitudinal fasciculus^31^, which predicted deterioration of social functioning throughout longitudinal follow-up. The SLF runs through the core of the white matter of the cerebral hemisphere and connects parieto-frontal network. The SLF with abnormal FA, AD and RD indicates disturbances in the fronto-parietal control network in ASPD patients. Note that the fronto-parietal control network is associated mainly with cognition control function^32^. Executive function (EF) reflects higher order cognitive control of thought, action, and emotions. Neuropsychological deficits in executive function were thought to contribute to severe antisocial and aggressive behavior^33^,^34^,^35^. The decrease of SLF connectivity may result in deficient cognitive, behavioral control, and mood processing, thus resulting in poor self-control ability, apathy, impulsivity and risk-taking behaviors of ASPD patients. The correlation with impulsivity or risky behaviors may better certify that point.

The corpus callosum (CC) connects the two cerebral hemispheres. Lesion studies of CC revealed its role in supporting sensory-motor functional integration, attention, language, inter-hemispheric transfer of associative learning, and emotional regulation^36^,^37^,^38^. The CC was also found to be involved in impulsive personalities^39^ and cognitive deficit^40^. It was believed to play an important role in understanding social situations and adopting appropriate social behavior^41^. The abnormalities of CC were found in the adolescents who engaged in dangerous behavior^42^. Hiatt and Newman previously suggested the impaired functioning of the corpus callosum in psychiatric offenders on the basis of prolonged inter-hemispheric transfer time^43^. In our study, the reduced AD in the corpus callosum of ASPD disorders may suggest that the functions predominated by the left hemisphere, including approach behavior and language processing, may be unmodulated by functions mediated mainly by the right hemisphere (e.g., behavioral inhibition and emotion processing) as postulated by Hiatt and Newman^43^. This proposed mechanism may also help explain the increased impulsivity and dangerous behavior in ASPD disorders. In our study, the finding that AD values of SCC were correlated with impulsivity may certify this point.

Bilateral anterior limbs of the internal capsule are known to have numerous cortical projections (including those from frontal and temporal cortices) to the thalamus, and then ultimately to the brainstem^44^. Deficits in the anterior limb of the internal capsule was involved in acquired sociopathy^45^. Furthermore, lesions of the anterior limb of the internal capsule were believed to lead to impairments in attention, perception and working memory^46^,^47^. The anterior limb of the internal capsule together with the anterior corona radiate have been postulated to contribute to attention impairments of alerting and conflict processing, respectively^48^. Disruption in these tracts may contribute to problems in adaptive response to contingencies in the physical and social environment, expressed in behavioral traits such as impulsivity, aggression, and failure to learn from aversive experiences, which are characteristics typically exemplified in ASPD individuals. In our study, the RD values of ACR show correlated with impulsivity or risky behaviors.

In this study, we also found a few regions with increased FA in the ASPD subjects compared to controls. Interestingly, the regions with FA increases in the ASPD subjects are mostly either located in the superficial white matter (WM) areas or near the superficial WM areas. The superficial WM areas are interleaved between the cortex and the deep white matter^49^. In our study, the superficial WM areas are the left and right middle frontal blade, respectively. As discussed above, the frontal gyri are important for ASPD behavioral control and emotional adjustment. The findings of FA increases in these regions in the ASPD subjects compared with controls may reflect the atypical neurodevelopmental processes underlying the antisocial and aggressive behaviors. Actually, the atypical neurodevelopmental processes in ASPD patients/violent offenders were also reported in the previous studies^50^,^51^. In our study, they may occur as compensatory of other tracts deficits during the development of persistent antisocial behavior.

In total, FA, AD and RD deficits in this study were found in many major white matter pathways mainly connecting the fronto-parietal control network and fronto-temporal network, and these pathways show correlated with impulsivity or risky behaviors. Our study *not only* further certifies the fronto-temporal brain abnormalities from the white matter microstructure, *but also* reveals the abnormality in the fronto-parietal control network. These biological deficits may interact with environment to create a “disconnection” underpinning numerous social, emotional, or cognitive deficits in ASPD patients. The microstructural properties of ASPD may have potential to reveal the neurobiological mechanisms under the appearances of impulsivity, aggression and callous mind associated with ASPD. Since our controls were recruited from the School for Youth Offender, it is necessary to study the alteration of ASPD patients by comparing with the “non-offender” subjects (as controls) from local communities. So we will continue our DTI study of ASPD by comparing with the “non-offender” subjects in the future, especially for the possible correlation between DTI and behavioral measures.

## Methods

### Participants

All volunteers were recruited from the School for Youth Offender of Hunan Province in China, where they received reformatory education for certain committed crimes, e.g., robbery and violent attacks. All young offenders were of legal age (age >18 years) to provide formal consent when taking part in the experiments, but under the legal age when entering the school. In this school, they received an “Enclosed-style” management with regular school hours daily. As a measure for ASPD diagnosis, two main steps were followed. First, an experienced psychiatrist used the Personality Diagnostic Questionaire-4+ (PDQ-4+) to test all volunteers in groups at the school. The residuals with ASPD scores equal to or above 4 score were sequentially tested using the structured clinical interview for DSM-IV (SCID-II) by two senior psychiatrists. The SCID-II is a diagnostic exam used to determine personality disorders. In this step, those were excluded if the ASPD disorders exhibited other personality disorder. Finally, 20 subjects were diagnosed with only ASPD, i.e., all ASPD disorders met both PDQ-4 criteria and SCID-II criteria for ASPD but without other personality disorder. We also recruited 23 healthy controls and they met *neither* PDQ-4 criteria *nor* SCID-II criteria for ASPD. One-way ANOVAs were performed on the demographics of the groups to test whether the two groups were well matched. The results show that the controls are matched to ASPD patients in age, education, and IQ (Table 3). Here, the IQ scores were obtained using Wechsler Adult Intelligence Scale.

Although ASPD subjects often have a past history of alcohol or substance misuse, we attempted to recruit subjects without history or current diagnosis of drugs or alcohol abuse. All subjects also had no chance to touch alcohol/drug for a minimum of 6 months prior to the brain MRI scan, i.e., they entered in the school for at least 6 months. All the subjects were all right-handed native Chinese speakers without history or current diagnosis of serious mental disorders, e.g., schizophrenia, depression or anxiety neurosis. While undergoing the MRI scans, each subject was accompanied by three instructors.

The Ethics Committee of the Third Xiangya Hospital of Central South University and the Ethics Committee of the School for Youth Offender of Hunan Province approved this study. After receiving a detailed description of the study, all the potential volunteers were free to accept or decline participation in the study. Written informed consents were obtained from all final participants. The whole procedure was carried out in accordance with the approved guidelines of Institutional Review Board (IRB).

### Behavioral Access

According to DSM-V, impulsivity is an important and central characteristic of ASPD. To measure the impulsivity of each subject, we used the Barratt Impulsiveness Scale (BIS-11)^52^, the most popular measure of impulsive personality traits. The BIS-11 is a 30-item questionnaire and each item was measured on a 4-point scale (rarely/never, occasionally, often, and almost always), with the higher summed scores indicative of higher levels of impulsivity^52^. The total scores of BIS-11 can range from 30 to 120. BIS-11, originally developed in the United States^52^, has been broadly used around the world with translations in more than 12 languages other than English^53^. BIS-11 is also a suitable tool for measuring impulsiveness in Chinese culture with high reliability and test-retest reliability^54^.

Following the questionnaire, we used the health-risk behavior inventory for Chinese adolescents (HBICA)^55^ to measure their risk behavior. HBICA can investigate adolescent risky behaviors in China and distinguish high-risk individual effectively. It has good reliability (0.66**–**0.76) and validity (0.77–0.86)^55^. The HBICA is a 33-item self-report questionnaire, where each item was measured on a 5-point response from 1 (never) to 5 (always). The total scores of HBICA range from 33 to 155, with the higher summed scores indicative of higher levels of risky behavior.

The result of one-way ANOVAs showed significant differences between ASPD subjects and controls. The ASPD subjects had higher impulsivity and more risky behavior (Table 3).

### Image Acquisition

All MRI data were obtained on a 3T Philips scanner located at the Third Xiangya Hospital of Central South University. The principal sequence of this study was a single-shot spin echo planar imaging sequence and DTI images were acquired with the following parameters: repetition time (TR) = 6619 ms, echo time (TE) = 86 ms, field of view (FOV) = 200 mm, matrix = 144 × 144, 2 averages, diffusion sensitizing orientations = 32 with b = 1000 s/mm^2^, and one with b = 0 s/m^2^, 60 axial slices with resolution = 1.67 × 1.67 × 2.5 mm^3^, acquisition time = 8 min 30 s. Images were inspected for motion artifact at the time of acquisition and scans were repeated as necessary. Images were also reviewed and discarded, if there were any pathological findings.

### Image Processing

All DTI data were processed using Panda v1.3.0^56^, a pipeline toolbox for analyzing brain diffusion images based on FSL tools^57^. Briefly, in the pipeline, the raw DTI images were first resampled to 2 × 2 × 2 mm^3^ resolution. Next, skull removal with the brain extraction tool (BET) was done to extract brain tissue for B = 0 image in each subject. Eddy current correction was applied to correct for distortion, head motion and eddy currents on the original DTI data by registering the DW images to the B = 0 image with an affine transformation. Diffusion metrics, i.e., FA, AD and RD, were then calculated for voxels within a mask created from the B0 image. These data were spatially normalized to a FMRI58 space template. Quantity control reports of resulting images were created. We reviewed these quality control files and discarded any volume with problems that could potentially skew the data, e.g., large motion artifacts or noise. For tract-based spatial statistics (TBSS), we first acquired a skeletonized image from the average FA of all the 43 FA images from 43 subjects. By using the threshold of 0.25 for FA value, the mean FA skeleton was thinned to identify the fiber pathways, which were consistent across subjects. Then, this thresholded FA skeleton image was projected to each subject’s standardized images, i.e., FA, AD and RD images. These skeletonized FA, AD and RD images were used in subsequent statistical analyses to test study hypotheses.

### Statistical Analyses

To compute group differences, voxel wise statistical analysis was performed on FA, AD and RD maps in ASPD subjects and controls across the entire cerebrum using Tract-Based Spatial Statistics (TBSS) version 1.2^58^. Relevant *t* contrasts were ASPD > Controls and ASPD < Controls. In this step, we used false discovery rate (FDR) for the multiple comparisons correction of the original statistical results. Primary group contrast results were presented if their *p* < 0.05 after FDR correction and the cluster size was larger than 50, when searching the entire skeleton.

To determine if significant differences in FA, AD and RD were associated with behavioral variations, we performed correlation analyses between impulsivity or risky behaviors and the regional DTI measures. First, each cluster identified at the first step was extracted from DTI measure maps accordingly for each subject. Second, the average FA, AD and RD values in each relevant cluster were calculated. Finally, the Pearson correlation coefficient was calculated between the DTI measures of each relevant cluster and the behavioral measure (*p* < 0.05, uncorrected).

## Additional Information

**How to cite this article**: Jiang, W. *et al*. Reduced White Matter Integrity in Antisocial Personality Disorder: A Diffusion Tensor Imaging Study. *Sci. Rep.* **7**, 43002; doi: 10.1038/srep43002 (2017).

**Publisher's note:** Springer Nature remains neutral with regard to jurisdictional claims in published maps and institutional affiliations.

## Figures and Tables

**Table 1:** Significant alterations in fractional anisotropy (FA) measurement of diffusion tensor imaging (DTI), in ASPD patients relative to control subjects.

cluster index	voxel size	region name	peak voxel			t value	p value
			x	y	z		
**Fractional anisotropy (FA)**							
ASPD < Control							
1	126	Superior longitudinal fasciculus L	−43	−3	10	−3.76	2.52E-04
2	123	Anterior limb of internal capsule/Anterior corona radiata/Inferior fronto-occipital fasciculus/R	18	42	−30	−3.07	1.83E-03
3	74	Retrolenticular part of internal capsule L	−33	−13	−13	−3.61	3.91E-04
4	72	Superior corona radiata/Superior longitudinal fasciculus L	−33	7	4	−3.95	1.41E-04
5	70	Middle frontal blade R	37	13	18	−3.35	8.34E-04
6	60	Inferior fronto-occipital fasciculus/Anterior corona radiata L	−20	40	−31	−3.23	1.17E-03
7	57	Inferior fronto-occipital fasciculus/Uncinate fasciculus R	35	13	−34	−3.92	1.55E-04
8	55	Post-central blade/Pariteo-temporal blade L	−45	−29	16	−3.66	3.34E-04
9	50	Fornix/Stria terminalis L	−25	2	−28	3.36	8.09E-04
ASPD > Control							
1	78	Corticospinal tract R	23	−22	28	3.08	1.76E-03
2	66	Superior frontal blade L	−15	7	36	4.15	7.57E-05
3	61	Middle frontal blade R	31	19	20	4.87	7.45E-05
4	59	Superior longitudinal fasciculus R	43	−5	14	3.65	3.50E-04
5	53	Inferior fronto-occipital fasciculus/Forceps major L	−30	−54	−17	3.45	6.32E-04
6	50	Superior frontal blade L	−16	31	29	3.96	1.36E-04

R: right; L: left.

**Table 2:** Significant alterations in axial diffusivity (AD), and radial diffusivity (RD) measurements of diffusion tensor imaging (DTI), in ASPD patients relative to control subjects.

cluster index	voxel size	region name	peak voxel			t value	p value
			x	y	z		
**Axial diffusivity (AD)**							
ASPD < Control							
1	516	Superior corona radiata/Superior longitudinal fasciculus/Posterior corona radiata L	−34	3	6	−3.80	2.18E-04
2	289	Splenium of corpus callosum/Body of corpus callosum	−16	−25	−9	−3.07	1.82E-03
3	231	Anterior corona radiata/Anterior limb of internal capsule L	−24	43	−20	−3.12	1.60E-03
4	185	Superior corona radiata R	19	3	21	−3.58	4.22E-04
5	177	Superior corona radiata/Posterior limb of internal capsule/Posterior corona radiata L	−25	1	−9	−4.03	1.08E-04
6	151	Pre-central blade/Superior frontal blade R	17	−4	37	−4.26	5.36E-05
7	118	Anterior corona radiata L	−20	50	9	−3.17	1.40E-03
8	95	Posterior corona radiata/Posterior thalamic radiation (include optic radiation) L	−30	−45	−4	−4.81	9.06E-06
9	82	Post-central blade/Pariteo-temporal blade L	−47	−19	15	−3.07	1.81E-03
10	75	Inferior frontal blade L	−35	14	19	−4.25	5.53E-05
11	71	Superior longitudinal fasciculus L	−45	−1	10	−3.47	5.82E-04
12	67	Anterior corona radiata L	−20	67	−15	−2.92	2.72E-03
13	66	Posterior corona radiata L	−20	−38	16	−3.55	4.59E-04
14	61	Superior longitudinal fasciculus R	34	−6	17	−3.50	5.38E-04
15	58	Fornix (cres)/Stria terminalis L	−21	−21	−13	−3.62	3.75E-04
16	58	External capsule L	−32	−3	−15	−3.88	1.75E-04
17	50	Superior corona radiata R	26	−26	21	−3.44	6.43E-04
**Radial diffusivity (RD)**							
ASPD > Control							
1	108	Superior longitudinal fasciculus R	48	−5	9	3.52	5.06E-04
2	74	Superior frontal blade R	11	30	33	3.65	2.69E-03
3	65	Anterior corona radiata/Inferior fronto-occipital fasciculus L	−19	42	−29	3.65	3.48E-04
4	62	Inferior longitudinal fasciculus/Temporal blade L	−46	2	−41	4.06	9.85E-05

R: right; L: left.

**Table 3:** Characteristics of the participants in this study.

	ASPD	Controls	P value
	(Mean ± SD)	(Mean ± SD)	
Number	20	23	—
Gender	20 males	23 males	—
Age (Years)	21.8 ± 3.2	22.1 ± 3.9	0.744
Education (Years)	8.3 ± 1.5	9.7 ± 0.8	0.653
IQ	95.2 ± 8.4	95.5 ± 8.6	0.927
BIS score	66.9 ± 4.7	61.8 ± 6.3	0.006
HBICA score	79.7 ± 13.7	54.7 ± 10.2	0.000

ASPD: Offenders with antisocial personality disorder.

BIS: Barratt Impulsiveness Scale.

HBICA: Health-Risk Behavior Inventory for Chinese Adolescents.
